# Impact of Diet, Nutrition and Lifestyle on Reproductive Health

**DOI:** 10.3390/nu18020227

**Published:** 2026-01-12

**Authors:** Paula Benny, Cuilin Zhang, Zhongwei Huang

**Affiliations:** 1 NUS Bia-Echo Asia Centre for Reproductive Longevity and Equality (ACRLE), National University of Singapore, Singapore 117597, Singapore; 2 Department of Obstetrics & Gynaecology, Yong Loo Lin School of Medicine, National University of Singapore, Singapore 117597, Singapore; 3 Global Centre for Asian Women’s Health, Yong Loo Lin School of Medicine, National University of Singapore, Singapore 117597, Singapore

This Special Issue on the “Impact of Diet, Nutrition and Lifestyle on Reproductive Health” highlights the important roles of diet [1], nutrition, and different lifestyle choices [2] in the human reproductive life- and healthspan.

Several studies in this Special Issue investigated methods targeting male and female infertility in different populations. Towards understanding mechanisms of female fertility, Maldonado-Carceles (contribution 1) and colleagues (EARTH study; Environment and Reproductive Health) showed that lycopene supplementation had a positive linear association with antral follicle count (AFC). Conversely, retinol intake, predominantly from dairy foods, was inversely correlated with AFC in women aged below 35. Mitsunami (contribution 2) and colleagues also showed that dietary intake of higher glycemic index was associated with higher AFC.

Towards understanding mechanisms of male fertility, a review by Michaelsen et al. (contribution 3) showed that certain supplements were able to improve single sperm parameters—in particular, zinc and folic acid improved sperm concentration; selenium, carnitine, and coenzyme Q10 improved motility; and alpha-lipoic acid improved normal morphology; while others, such as vitamin D, vitamin E, and omega-3 fatty acids, showed no improvement in sperm parameters, prompting the authors to suggest that larger and more well-conducted experimental studies that focus on specific dietary supplements and include pregnancy-related outcomes are needed. In addition, a review by Win (contribution 4) and colleagues highlighted the effects of sugar-sweetened beverages on male fertility through changes in critical sperm parameters such as sperm count, motility, and morphology.

Two studies attempted to target both male and female partners to optimize preconception health towards a healthier pregnancy. Nizamani (contribution 5) and colleagues showed that tailored dietary and physical activity interventions were able to reduce the number of sedentary hours, which could ultimately reduce metabolic syndrome risk. Lopez (contribution 6) and colleagues showed that probiotic interventions increased pregnancy success in couples with unexplained infertility by improving both vaginal and seminal immunological profiles prior to in vitro fertilization and affecting reproductive success.

In a multi-ethnic pregnancy cohort, Miller (contribution 7) and colleagues showed that increased carbohydrate consumption and improved diet quality may be associated with beneficial vaginal microbial composition, reducing pregnancy complications such as preeclampsia and fetal growth restriction. Furthermore, de Siqueira (contribution 8) and colleagues analyzed mammary epithelial cell cultures to explore the effects of serum amyloid A protein in breast cells and breast milk to unravel the mechanisms governing milk production and breastfeeding patterns.

In addition to basic and translational studies, population health studies, such as that of Craciun (contribution 9) and colleagues, showed that dietary patterns (e.g., prudent, Western, and unhealthy patterns) were associated with social determinants of health and lifestyle components in men and women of reproductive age, highlighting the need for personalized educational interventions to effectively target individuals in the higher-risk categories to improve overall health—particularly for individuals of reproductive age. Similarly, in an Asian context (India), Sunu (contribution 10) and colleagues studied the importance of accounting for micronutrient deficiencies and undernutrition owing to the socio-cultural characteristics of indigenous tribes, as well as the importance of developing a culturally sensitive nutrition intervention model for improving women’s health in various communities. Finally, Szulinska (contribution 11) and colleagues reported on the benefits of nutrition counseling in a European population (e.g., optimizing protein intake and plant-based nutrition sources), which may improve body composition and potentially improve fertility outcomes in women undergoing fertility treatments.

Finally, the Editor’s choice article from Sziva (contribution 12) and colleagues showed that in a preclinical polycystic ovary syndrome (PCOS) model, early interactions of hyperandrogenism and vitamin D deficiency could separately lead to impaired vascular reactivity and ovulatory dysfunction in fertile females, thus providing new information on the biological mechanisms governing ovarian health and the importance of nutrition.

In summary, this Special Issue collects 12 articles contributed by authors globally, comprising evidence-based reviews, clinical translational research, and original laboratory data, providing novel insights into the role of lifestyle, diet, and nutrition in maintaining and promoting reproductive health in both men and women, which will naturally have an impact on the health of future generations.
